# Supplementary material for: Effect of Zanthoxylum bungeanum essential oil on rumen enzyme activity, microbiome, and metabolites in lambs
Source: PLoS One. 2022 Aug 5;17(8):e0272310. doi: 10.1371/journal.pone.0272310 (PMC9355197; doi:10.1371/journal.pone.0272310)
Supplement: S1 Table — (DOCX) [file pone.0272310.s001.docx]

Table1 Sample sequencing data statistics

| Sample | Tags | Clean_tags | Effective(%) | OTUs |
| --- | --- | --- | --- | --- |
| ALW.1 | 139384 | 129528 | 92.93 | 2218 |
| ALW.2 | 138625 | 131288 | 94.71 | 2343 |
| ALW.3 | 133207 | 126499 | 94.96 | 2210 |
| ALW.4 | 133491 | 125780 | 94.22 | 2015 |
| ALW.5 | 147832 | 138615 | 93.77 | 2231 |
| BLW.1 | 132081 | 125284 | 94.85 | 2088 |
| BLW.2 | 143810 | 133972 | 93.16 | 2127 |
| BLW.3 | 140335 | 132131 | 94.15 | 2177 |
| BLW.4 | 131510 | 122813 | 93.39 | 2061 |
| BLW.5 | 140550 | 135512 | 96.42 | 2197 |
| CLW.1 | 131003 | 117434 | 89.64 | 2431 |
| CLW.2 | 132557 | 123154 | 92.91 | 2158 |
| CLW.3 | 141856 | 132772 | 93.60 | 2237 |
| CLW.4 | 133941 | 121991 | 91.08 | 2319 |
| CLW.5 | 136425 | 128927 | 94.50 | 2128 |
| DLW.1 | 133353 | 125429 | 94.06 | 2238 |
| DLW.2 | 133049 | 123809 | 93.06 | 2259 |
| DLW.3 | 129079 | 122693 | 95.05 | 2173 |
| DLW.4 | 133058 | 125388 | 94.24 | 2361 |
| DLW.5 | 142416 | 129887 | 91.20 | 2062 |
| Total | 2727562 | 2552906 |  | 3678 |
